# Synthesis and Biological Evaluation of Ezetimibe Analogs as Possible Cholesterol Absorption Inhibitors

**DOI:** 10.2174/157018011795906776

**Published:** 2011-07

**Authors:** Yubin Wang, Haiqian, Wenlong Huang, Huibin Zhang, Jinpei Zhou

**Affiliations:** a School of Pharmaceutical Sciences, Nanjing University of Technology, NO.5 Xinmofan Road, Nanjing 210009, China; b Department of Pharmacology, China Pharmaceutical University, 24 Tongjiaxiang, Nanjing 210009, China

**Keywords:** 2-Azetidinone derivatives, Enantiomerically pure, Cholesterol absorption inhibition.

## Abstract

In order to investigate the SAR of Ezetimibe analogs for cholesterol absorption inhibitions, amide group and electron-deficient pyridine ring were introduced to the C-(3) carbon chain of Ezetimibe. Eight new derivatives of the 2-azetidinone cholesterol absorption inhibitors have been synthesized, and all of them were enantiomerically pure. All the new compounds were evaluated for their activity to inhibit cholesterol absorption in hamsters, and most of them showed comparable effects in lowering the levels of total cholesterol in the serum.

## 1 INTRODUCTION

Atherosclerotic coronary heart disease (CHD) has been the major cause of death and cardiovascular morbidity in the world [1]. The prominent risk factor associated with CHD was the elevation of serum cholesterol levels [2]. Well established clinical treatment for CHD has focused on life style changes and the reduction of serum cholesterol. These reductions have been shown to correlate strongly with the decrease of CHD mortality and the reversal of atherosclerosis as evidenced by the regression of occlusion of coronary arteries [3]. Pharmacologically these reductions have focused on the use of “statins” or HMG-CoA reductase inhibitors to affect both the biosynthesis of cholesterol and clearance mechanisms [4]. The other major contributor to serum cholesterol is from exogenous (dietary) or intestinal sources (enterohepatic circulation of biliary cholesterol). Blocking intestinal sources of cholesterol represents a scientifically and pharmacologically interesting mechanism for affecting serum cholesterol as it complements existing therapies in the clinic [5].

**Fig. (1):**
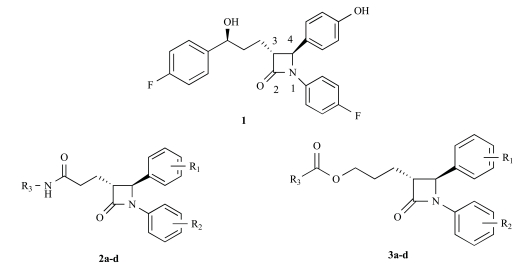
The chemical structures of ezetimibe and analogs.

Ezetimibe(**1**) (Fig.**1**), which was approved in late 2002 for use either alone or in combination with a statin, was the only example to date of a drug that involves inhibition of intestinal cholesterol absorption [6]. A recent report from the *Schering-Plough *Research Institute has described the discovery of *Niemann-Pick *C1 Like 1 (NPC1L1) protein as critical for the intestinal absorption of cholesterol. Knockout mice lacking the NPC1L1 gene showed markedly reduced cholesterol absorption and were no longer sensitive to further reduction of cholesterol absorption by ezetimibe. Thus NPC1L1 lies in the ezetimibe sensitive pathway for cholesterol absorption, making it a likely candidate for the target of ezetimibe [7].

The reported structure-activity relationships (SAR) studies revealed that the 2-azetidinone was required for activity, the C-(3) sidechain was optimal at three linking atoms bearing a pendent aryl group and the C-(4) aryl residue was required and was optimally substituted with a polar moiety at the *para* position, the N-aryl ring was also required and was tolerant of a wide variety of substitutions [8-10]. It is known that bioisosterism is an important lead modification approach that has been shown to be useful to attenuate toxicity or to modify the activity of a lead, and many have a significant role in the alteration of pharmacokinetics of a lead. In order to investigate the effect of the polarity of the C-(3) sidechain on cholesterol absorption inhibition, we used bioisosteric interchange and introduced amide group to the C-(3) carbon chain in compounds **2a-d**, increasing the polarity of C-(3) sidechain. The relative configuration at C-(3) and C-(4) of compounds **2a-d** were all (*3R, 4S*)*.* On the other hand, another chemical modification of **1 **in our research was electron-deficient pyridine ring and ester group to the C-(3) carbon chain in compounds **3a–d**. As a result, the ezetimibe analogs **2a-d **and **3a-d **(Fig. **1**) were designed, synthesized and their ability to inhibit cholesterol absorption was evaluated [11].

## 2 RESULTS AND DISCUSSION

### 2.1 Chemistry

**Scheme. 1:**
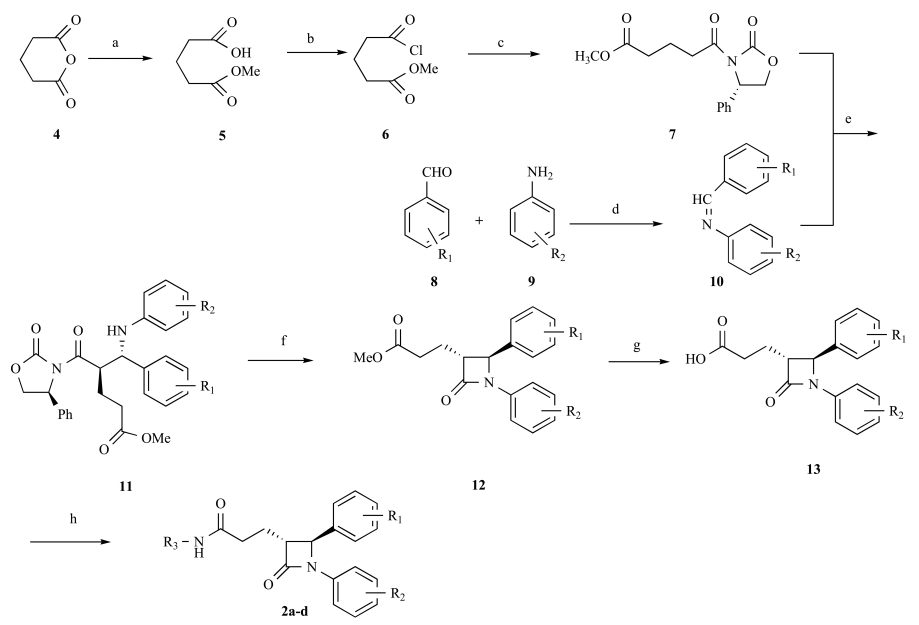
Reagents and conditions: (a) CH_3_OH, reflux, 1h; (b) SOCl_2_, reflux, 2h; (c) (S)-(+)-4-phenyl-2-oxazolidinone, Et_3_N CH_2_Cl_2_, r t, 5h; (d) iPrOH reflux, 2h; (e) TiCl_4_, DIPEA, CH_2_Cl_2_, -30- 40 °C, 4h; (f) BSA,TBAF, toluene, 40-50 °C, 4h; (g) LiOH, THF/H2O, r t, overnight; (h) substituted aromatic amine or aliphatic amine, DCC/DMAP, CH_2_Cl_2_, r t, overnight.

The synthetic route to **2a-d **is summarized in Scheme **1** [12]**. **Refluxing glutaric anhydride (**4**) with an equivalent amount of anhydrous MeOH afforded monomethyl glutarate (**5), **and treatment of **5** in refluxing SOCl_2_ yielded methyl 4-(chloroformyl)butyrate (**6**) in excellent yield 84.9% without further purification. Reactions of methyl 4-(chloroformyl) butyrate (**6**) with (S)-(+)-4-phenyl-2-oxazolidinone in the presence of Et_3_N in anhydrous CH_2_Cl_2_ at room temperature gave intermediate **7. **Reaction of a substituted aromatic aldehyde **8** with a substituted aromatic amine **9** in refluxing isopropyl alcohol gave imines **10**. Then, intermediate** 7** was treated with TiCl_4_, Hünig’s base, and the corresponding imine **10** to give the intermediate β-aminoxazolidinone **11**. The major diastereomer was purified to homogeneity by crystallization and then cyclized in two steps by first silylation with bistrimethylsilylacetamide (BSA) followed by treatment with a catalytic amount of tetrabutylammonium fluoride (TBAF) to get a single enantiomerically pure intermediate **12**. Then hydrolysis and amidation of **12 **led to enantiomerically pure 2-azetidinone analogs **2a-d**.

**Fig. (2):**
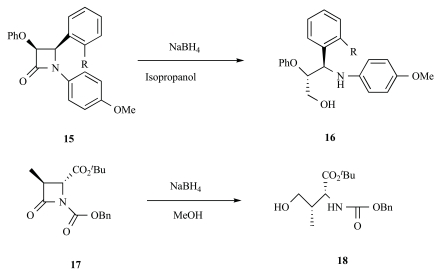
Reductive opening of β-lactam ring.

**Scheme. 2:**
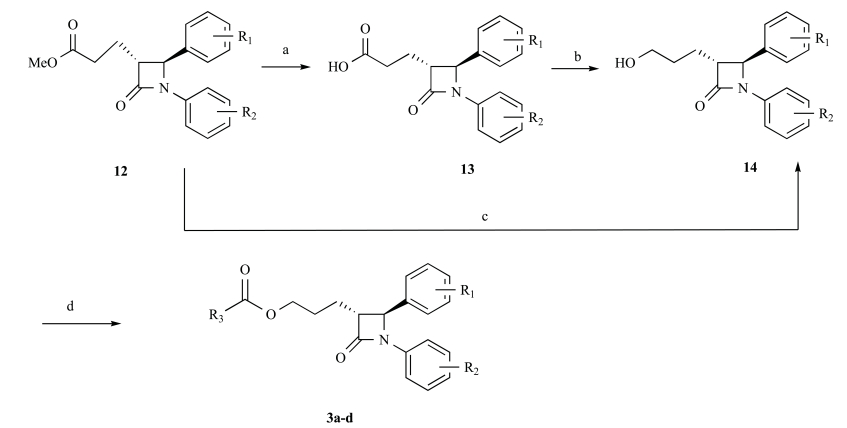
Reagents and conditions: (a) LiOH, THF/H_2_O, r t, overnight; (b) NaBH_4_, I_2_, THF, reflux, 6h; (c) NaBH_4_, THF, reflux, 24h; (d) substituted aromatic acid, DCC/DMAP, CH_2_Cl_2_, r.t, overnight.

The synthesis of target compounds **3a-d** is summaried in Scheme **2**. The intermediate **12 **was used as started material. Preparation of alcohol **14** was the key step in synthesis of target compounds because the β-lactam ring would be opened by reductive reagents. According to literature, β-lactam compound **15** and **17** could undergo reductive opening in the presence of sodium borohydride in methanol or isopropanol (Fig. **2**) [13-14]. At beginning, methyl ester **12** was reduced by NaBH_4 _in THF as solvent at room temperature for 5h. Unfortunately neither the ester-reducing product nor ring-opening product was obtained, even when the reaction was warmed to reflux. After that the solvent was changed to methanol according to literature^12^. It was observed that methyl ester **12** was transformed to alcohol **14** in yield of 12% along with the starting material in majority. The key intermediate **14** was not acquired from methyl ester **12 **in good yield, but these results were also encouraging to us, since it was found that the β-lactam ring in our compounds were more stable comparatively, at least upon treatment with NaBH_4._ It was known that reduction of carboxylic acids to alcohols was an important tansformation in synthetic organic chemistry and several methods were available for this purpose such as NaBH_4_-I_2 _in THF. So methyl ester **12** was first hydrolyzed by LiOH-H_2_O to give acid **13** in almost quantitative yield, and then reduced to obtain alcohol **13**. In a typical procedure [15], the carboxylic acid **13** was added slowly to the suspension of NaBH_4_ in THF and the mixture stirred until gas evolution ceased. Iodine in THF was then added slowly at room temperature and then the contents were warmed to reflux for 1.5-2 h. The reaction was terminated at once the starting material disappeared basically. After the usual workup, the alcohol **14** was obtained in ideal yield. Finally the reaction of **14** with substituted aromatic acid in the presence of DCC/DMAP in anhydrous CH_2_Cl_2_ at room temperature gave 2-azetidinone derivatives **3a–d** in good yields (60.8–66.4%).

The structures and spectral characteristics of the target compounds **2a-d** and **3a-d** were mentioned in Table **1** and Table **2**.

### 2.2 Biological Studies

Cholesterol absorption inhibition was assessed in orally dosed, cholesterol-fed hamsters as reported in literature [16]. The result is presented in the Table **3**. As can be seen from the data, most of the new compounds demonstrated moderate effect in lowering the total cholesterol in serum, especially compound **2c 2d 3a**and** 3b**, although their potency was still somewhat below that of ezetimibe. Compound **2a** and **2b** have no effect in lowering the total cholesterol in serum. It was also found that **3a** and **3b **could raise high-density lipoprotein cholesterol (HDL-C) levels markedly. This activity may be good for prevention and treatment of CHD. The current work suggests that the amide group in the C-(3) side chain was not critical for the cholesterol absorption inhibition activity and a pendent aryl group at the C-(3) sidechain was required. From the activity of **3a-d **in raising the HDL-C in serum, it was suggested that nicotinic acid may be crucial for raising high-density lipoprotein cholesterol. These SAR trends may provide insights into the further design of novel cholesterol absorption inhibitors.

## 3 CONCLUSIONS

In an effort to understand the SAR around cholesterol absorption inhibition, eight 2-azetidinone derivatives were synthesized and all of them were enantiomerically pure. Their cholesterol absorption inhibition activities were evaluated. 

Most of them showed comparable effects in lowering the levels of total cholesterol in the serum. These information could be valuable for further investigation of SAR and will be useful in later research of cholesterol absorption inhibitors.

## Figures and Tables

**Table 1:** Structures, Yields, Melting Points and of the Target Compounds

Compd.^a^	R_1_	R_2_	R_3_	Yield, %	mp, °C
**2a**	3,4-dioxolmethylene	4-Me	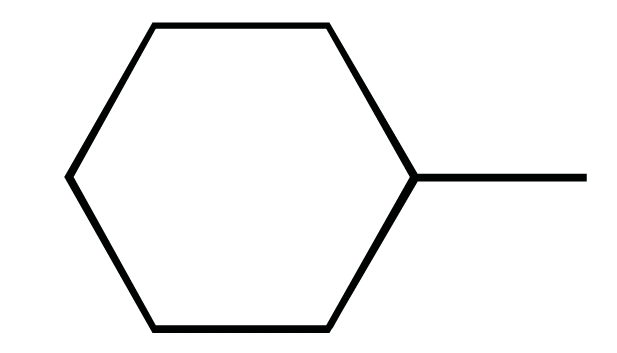	53.6	115－117
**2b**	3,4-dioxolmethylene	4-Me	n-C_3_H_7_	41.8	235-237
**2c**	3,4-dioxolmethylene	4-Me	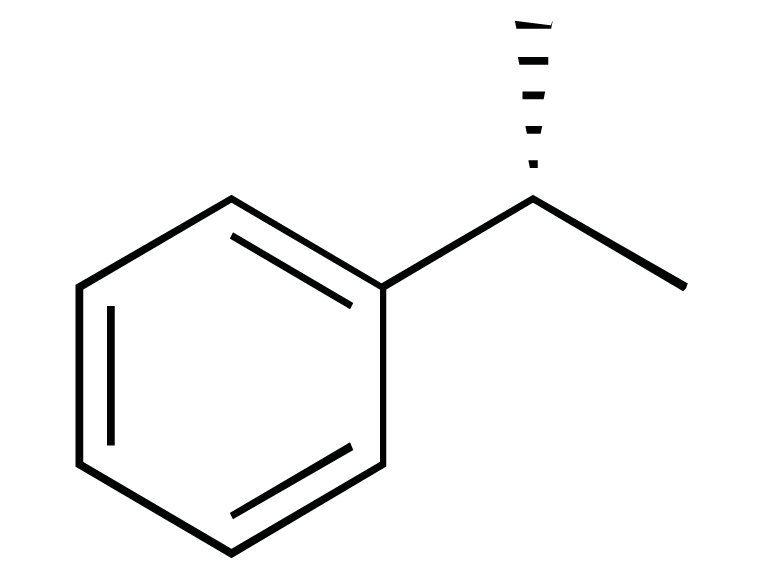	62.4	155-157
**2d**	4-OMe	4-OMe	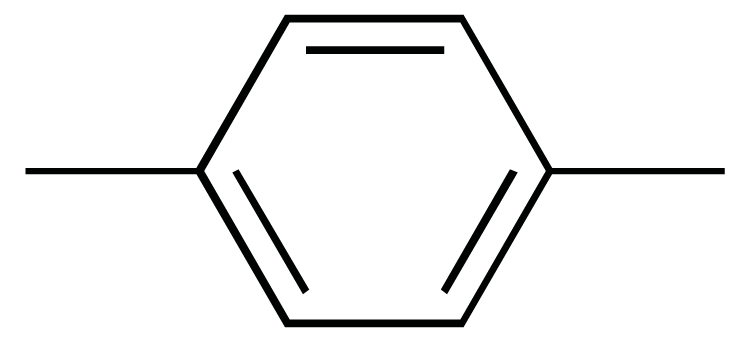	65.0	oil
**3a**	3,4-dioxolmethylene	4-Me	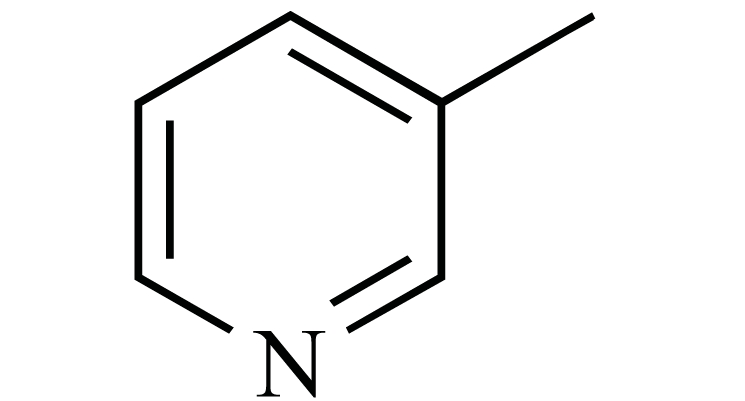	52.1	98-101
**3b**	3,4-dioxolmethylene-6-bromo-	4-Me	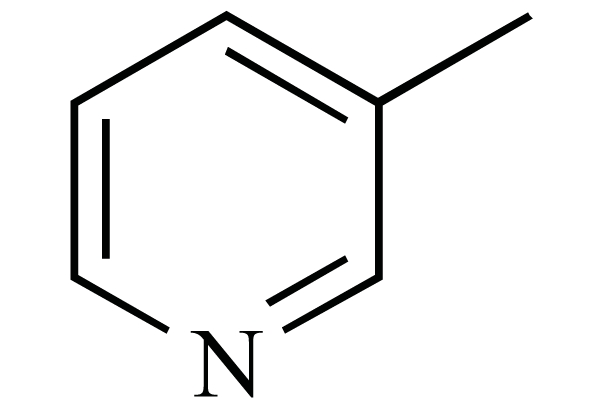	42.6	oil
**3c**	3,4-dioxolmethylene	4-Me	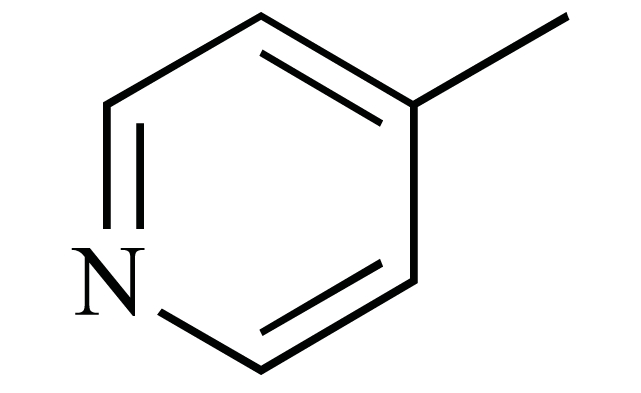	52.3	120-122
**3d**	3,4-dioxolmethylene	4-Me	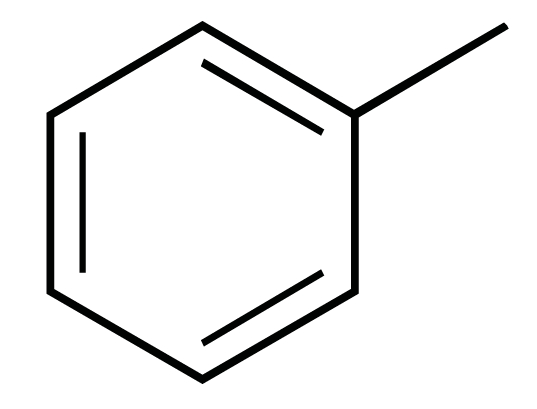	47.3	118-120

**Table 2:** Spectral Data of the Target Compounds

Compound	Spectral Data
**2a.**	^1^HNMR (CDCl_3_): 1.43-1.96 (m,10H, -(CH_2_)_5_-), 2.03-2.23 (m, 2H, -CH_2_-CH_2_-), 2.32 (s, 3H, Me), 2.52-2.65 (m**,** 2H, -CH_2_-CH_2_-), 3.06-3.11 (m, 1H, -CH-CH-), 4.60 (s, 1H, -CHN-), 3.66-3.68(m**,** 1H, -CH-), 5.94 (2H, s, -OCH_2 _O-), 6.77-6.87 (m, 3H, Ar-H), 7.03-7.17 (dd, 4H, Ar-H, J = 8.1Hz); MS (70eV) m/z: 435 ([M+H]^+^)。Anal.calc. for: C_26_H_30_N_2_O_4_ (434.22): C 71.53 H 7.14 N 6.69; found : C 71.87 H 6.96 N 6.45; [α]= -11.4 (C=0.33, D:589nm, T=20°C, solv:MeOH)
**2b**	^1^HNMR (CDCl_3_): 0.98-1.03 (m, 3H, -CH_2_CH_2_CH_3_), 1.43-1.64 (m, 2H, -CH_2_CH_2_CH_3_) 2.03-2.12 (m, 2H, -CH_2_-CH_2_-),2.35 (s, 3H, Me), 2.48-2.69 (m**,** 2H, -CH_2_-CH_2_-), 3.04-3.19(m**,** 2H, -CH_2_-), 3.24-3.43 (m, 1H, -CH-CH-), 4.87 (s, 1H, -CHN-), 5.94 (2H, s, -OCH_2 _O-), 6.57-6.67 (m, 3H, Ar-H), 6.95-7.18 (dd, 4H, Ar-H, J = 7.8Hz); MS (70eV) m/z: 417 ([M+H]^+^); Anal.calc. for: C_23_H_26_N_2_O_4_ (394.19): C 70.29 H 6.52 N6.97; found : C 70.03 H 6.64 N 7.10; [α]= -11.4 (C=0.055, D:589nm, T=20°C, solv:MeOH)
**2c**	^1^HNMR (CDCl_3_): 1.59－1.73(m, 3H, -CH_3_), 1.92-2.08 (m, 2H, -CH_2_-CH_2_-),2.29 (s, 3H, Ar-Me), 2.39-2.49 (m**,** 2H, -CH_2_-CH_2_-), 2.86-2.89 (m, 1H, -CH-CH-), 3.48-3.51 (m, 1H, -CHN-), 5.95 (2H, s, -OCH_2 _O-), 6.56 (s, 1H, Ar-H), 7.01 (s, 1H, Ar-H), 7.14-7.30(m, 9H, Ar-H); MS (70eV) m/z: 557, 559 ([M+H]^+^); Anal.calc. for: C_28_H_28_N_2_O_4_ (456.20): C 73.54 H 6.33 N6.27; found : C 73.66 H 6.18 N 6.14; [α]= -19.8 (C=0.185, D:589nm, T=20°C, solv:MeOH)
**2d**	^1^HNMR (CDCl_3_): 2.31(s, 3H, -CH_3_), 2.24-2.30(m, 2H, -CH_2_-CH_2_-), 2.59-2.64 (m, 2H, -CH_2_-CH_2_-), 3.09-3.14 (m, 1H, -CH-CH-), 3.78 (s, 6H, -OCH_3_), 4.66 (d, J = 2.0Hz, 1H, -CHN-), 6.76-6.85 (m, 4H, Ar-H), 7.19-7.27 (m, 4H, Ar-H), 7.28-7.40 (m, 4H, Ar-H), 7.79 (s, 1H, -NH-CO-); MS (70eV) m/z: 445.4([M+H]^+^); Anal.calc. for C_27_H_28_N_2_O_4_ (444.20): C 72.73 H 6.49 N 6.41; found : C 72.95 H 6.35 N 6.30; [α]= +13.3 (C=0.34, D:589nm, T=20°C, solv:MeOH)
**3a**	^1^HNMR (CDCl_3_,300MHz)δ: 1.96-2.17 (4H, m, -CH_2_-CH_2_-), 2.26 (s, 3H, -CH_3 _), 3.11 ( 1H, m, -CH-CO), 4.38-4.40 (2H, t, -CH_2_OCO, J=5.4Hz), 4.57(1H, d, -CHN, J=2.1Hz), 5.95 (2H, s, -OCH_2 _O-), 6.77-6.85 (3H, m, Ar-H), 7.034-7.191 (4H, dd, Ar-H, J=8.4Hz), 7.35-7.42 (1H, m, Py –H^5^), 8.27-8.30 (1H, d, Py –H^4^, J=8.4Hz), 8.70-8.83 (1H, m, Py –H^6^), 9.21 (1H, s , Py –H^2^); MS (70eV) m/z : [M+H]^+ ^445, [M+Na]^+ ^467, [M+K]^+ ^483; Anal.calc. for: C_26_H_24_N_2_O_5_ (444.17): C 70.53 H 5.27 N6.19; found : C 70.26 H 5.44 N 6.30; [α]= -15.6 (C=0.215, D:589nm, T=20°C, solv:MeOH)
**3b**	^1^HNMR (CDCl_3_,300MHz)δ: 2.16 (4H, m, -CH_2_-CH_2_-), 2.28 (s, 3H, -CH_3_ ), 3.10 ( 1H, m, -CH-CO), 4.43 (2H, s, -CH_2_OCO), 5.12 (1H, d, -CHN, J=2.1Hz), 5.96 (2H, s, -OCH_2 _O-), 6.72 (1H, s, Ar-H), 7.03-7.16 (5H, m, Ar-H), 7.37 -7.41(1H, m, Py –H^5^), 8.27 (1H, d, Py –H^4^, J=8.4Hz), 8.78 (1H, s, Py –H^6^), 9.20 (1H, s , Py –H^2^); MS (70eV) m/z : [M+H]^+ ^523.1, 525.1; Anal.calc. for: C_26_H_23_BrN_2_O_5_ (522.08): C 60.02 H 4.21 N5.15; found : C 59.67 H 4.43 N 5.35;
**3c**	^1^HNMR (CDCl_3_,300MHz)δ: 1.90-2.12 (4H, m, -CH_2_-CH_2_-), 2.27 (3H, s, -CH_3 _), 3.12 ( 1H, s, -CH-CO), 4.40 (2H, s, -CH_2_OCO), 4.58(1H, s, -CHN), 5.95 (2H, s, -OCH_2 _O-), 6.77-6.86 (3H, m, Ar-H), 7.04-7.191 (4H, dd, Ar-H, J=8.1Hz), 7.82-7.84 (2H, d, Py –H, J=4.5Hz), 8.79 (2H, s , Py –H); MS (70eV) m/z : [M+H]^+ ^445, [M+Na]^+ ^467, [M+K]^+ ^483; Anal.calc. for: C_26_H_24_N_2_O_5_ (444.17): C 69.91 H 5.68 N6.41; found : C 70.26 H 5.44 N 6.30; [α]= -25.2 (C=0.17, D:589nm, T=20°C, solv:MeOH)
**3d**	^1^HNMR (CDCl_3_,300MHz)δ: 1.94-2.12 (4H, m, -CH_2_-CH_2_-), 2.27 (3H, s, -CH_3 _), 3.12 ( 1H, s, -CH-CO), 4.35－4.37 (2H, d, -CH_2_OCO, J=5.4Hz), 4.58(1H, s, -CHN), 5.94 (2H, s, -OCH_2 _O-), 6.77-6.86 (3H, m, Ar-H), 7.04-7.195 (4H, dd, Ar-H, J=8.4Hz), 7.41-7.46 (2H, m, Ar-H), 7.54-7.58 (1H, m, Ar-H), 8.00-8.03 (2H, d, Ar-H, J=7.8Hz); MS (70eV) m/z : [M+H]^+ ^444, [M+Na]^+^466, [M+K]^+ ^482; Anal.calc. for: C_27_H_25_NO_5_ (443.17): C 73.34 H 5.47 N3.03; found : C 73.12 H 5.68 N 3.16; [α]= -28.3 (C=0.075, D:589nm, T=20°C, solv:MeOH)

**Table 3:** Cholesterol Absorption Inhibiton of New Analogs and Reference Compounds in Orally Dosed Seven Day Cholesterol Fed Hamsters

Compd.^a^	TC^b^(% reduction)	HDL-C^c^(% increase)
**2a**	NE^d^	NE^d^
**2b**	NE^d^	NE^d^
**2c**	27.3*	24.9
**2d**	28.1*	21.7
**3a**	39.8*	32.6*
**3b**	31.9*	28.4*
**3c**	26.7	19.2
**3d**	20.3	25.9
Ezetimibe	48.7**	37.3**

** P < 0.01

* P < 0.05

a 6-8 hamsters per group; Dose: 50 mg/kg.

b Reduction of total cholesterol comparing to the one in animals fed by high-cholesterol diets.

c Increase of HDL-C comparing to the one in animals fed by high-cholesterol diets.

d NE: no effect.
